# RNA remarkably promotes HIV-1 protease fast turnover for NCp15 processing in mild acidic conditions leading to condensation of HIV-1 nucleocapsid

**DOI:** 10.1186/1742-4690-10-S1-P61

**Published:** 2013-09-19

**Authors:** Sebastien Lyonnais, Laure Dufau, Michele Reboud-Ravaux, Roland Marquet, Jean-Christophe Paillart, Jose Maria Gatell, Rob Gorelick, Carine Tisné, Gilles Mirambeau

**Affiliations:** 1 IDIBAPS, Barcelona, Spain; 2 UPMC, Paris, France; 3 IBMC - CNRS - Univ. de Strasbourg, Strasbourg, France; 4 ACVP - SAIC - NCI Frederick, Frederick, USA; 5 Université Paris Descartes, CNRS, Paris, France

## 

During HIV-1 maturation, the step driven by its protease (PR) controling RNA condensation remains poorly documented.

Our methodology, mainly inspired by B. M. Alberts’ concept of macromolecular machines, combines biochemical and biophysical approaches with purified components. They provide a clear *in vitro* demonstration that HIV-1 RNA behaves within the HIV-1 particle as an up-regulator of PR. The resulting fast processing of RNA-bound nucleocapsid protein (NC) both in N- and C-termini from its Gag precursor clearly leads to RNA condensation. The critical step consists of the secondary cleavage releasing the Gag C-terminal p6 domain from the NCp15 intermediate. Remarkably, such processing is optimal in more physiological conditions than classically used for *in vitro* HIV-1 PR assay, thus allowing a useful protection of the crucial NC zinc fingers. The related mechanism implies PR sequestration by clusters of NCp15 assembled along the RNA chains, highlighting a fast condensation of RNA:NC ribonucleoproteic complexes as an opportune step within the overall process of maturation, prior to the conical capsid reassembly.

These data support a new biological paradigm of a protease dramatically controlled by a RNA molecule to optimize its action changing the targeted nucleoprotein architecture from an assembly mode to a functional one.

